# Distribution of pathogens and antimicrobial resistance in ICU-bloodstream infections during hospitalization: a nationwide surveillance study

**DOI:** 10.1038/s41598-021-95873-z

**Published:** 2021-08-19

**Authors:** Rami Sommerstein, Lauro Damonti, Jonas Marschall, Stephan Harbarth, Michael Gasser, Andreas Kronenberg, Niccolò Buetti

**Affiliations:** 1 Department of Infectious Diseases, Bern University Hospital, Bern, Switzerland; 2 Infection Control Programme, University of Geneva Hospitals and Faculty of Medicine, Geneva, Switzerland; 3 Swiss Centre for Antibiotic Resistance (ANRESIS), Institute for Infectious Diseases, University of Bern, Bern, Switzerland; 4 INSERM, IAME, University of Paris, Paris, France

**Keywords:** Antimicrobials, Clinical microbiology, Bacterial infection

## Abstract

Changing microorganism distributions and decreasing antibiotic susceptibility over the duration of hospitalization have been described for the colonization or infection of selected organ systems. Few data are available on bacteremias in the intensive care unit (ICU) setting. We conducted a nationwide study on bloodstream infection (BSI) using data from the Swiss Centre for Antibiotic Resistance (ANRESIS). We analyzed data on BSI detected in the ICU from hospitals that sent information on a regular basis during the entire study period (2008–2017). We described specific trends of pathogen distribution and resistance during hospitalization duration. We included 6505 ICU- BSI isolates from 35 Swiss hospitals. We observed 2587 possible skin contaminants, 3788 bacteremias and 130 fungemias. The most common microorganism was *Escherichia coli* (23.2%, 910), followed by *Staphylococcus aureus* (18.7%, 734) and enterococci (13.1%, 515). *Enterococcus* spp (p < 0.0001) and *Candida* spp (p < 0.0001) increased in proportion, whereas *E. coli* (p < 0.0001) and *S. aureus* (p < 0.0001) proportions decreased during hospitalization. Resistances against first- and second-line antibiotics increased linearly during hospitalization. Pathogen distribution and antimicrobial resistance in ICU-BSI depends on the duration of the hospitalization. The proportion of enterococcal BSI, candidemia and resistant microorganisms against first- and second-line antibiotics increased during hospitalization.

## Introduction

Intensive care unit acquired bloodstream infection (ICU-BSI) is a common healthcare-associated infection and is associated with high mortality^1–3^. Little is known on the relationship between the hospitalization duration and the epidemiology of ICU-BSI. Decreasing antibiotic susceptibility with increasing length of hospital stay has previously been illustrated for the infection of selected organ systems^4–6^. To our knowledge, the length of hospital stay and its impact on the ICU-BSI epidemiology was never specifically assessed in a large multi-centric cohort. Only few authors have investigated this question for bacteremia in the non-ICU setting^7,8^. Our objective was to describe distribution of pathogens and antimicrobial resistance in ICU-BSI according to the duration of hospitalization using a large national microbiological database.

## Material and methods

### Design and data collection

We conducted a nationwide, observational study on BSI using data from the Swiss Centre for Antibiotic Resistance (ANRESIS) data from 1st January 2008 to 31st December 2017. ANRESIS regularly receives information on all positive blood cultures from 30 Swiss microbiology laboratories, some of them collecting data from several hospitals^8^. Hospitals are distributed across the country and representing at least 80% of annual hospitalization days^9^. Accordingly, we analyzed data of BSIs from those Swiss hospitals that sent information on a regular basis (i.e., without major fluctuations in reporting) during the entire study period. Only isolates sampled in the ICU from Swiss hospitals sending information on hospital length of stay at time of sampling were included. In order to remove any bias introduced by an individual patient’s resistance evolution, only the first isolate of a species per patient was eligible for this analysis: duplicates (i.e., the same microorganism identified in subsequently collected blood samples) were excluded. Moreover, we restricted the dataset to pathogens that occurred > 10 times during the study period (i.e., belonging to predominant and/or relevant species). We used the term “bloodstream infection” (BSI) instead of “bacteremia and fungemia” throughout the text^10^.

### Microbiological data and resistance

Species identification and antimicrobial susceptibility testing are performed in local laboratories according to European Committee on Antimicrobial Susceptibility Testing (EUCAST, https://eucast.org) or Clinical and Laboratory Standards Institute (CLSI, https://clsi.org) guidelines. All laboratories are participating in at least one external quality program of either the Swiss quality control program issued by the Institute for Medical Microbiology, University of Zürich (http://www.imm.uzh.ch/services/qc.html), or the National External Quality Assessment Service (NEQAS; www.uknegas.org.uk).

Resistant isolates were defined as those who were resistant or displayed intermediate susceptibility against the antibiotic tested. Resistance against *first-line* antibiotics was defined as resistance against amoxicillin for enterococci, ceftriaxone and/or amoxicillin/clavulanic acid for Gram-negative microorganisms and oxacillin for *Staphylococcus aureus*^8^. All non-fermenting Gram-negative bacteria were considered as resistant to *first-line* antibiotics as detailed above. Resistance against *second-line* antibiotics was defined as vancomycin resistance for Gram-positive and carbapenem resistance for Gram-negative microorganisms. Fungal pathogens were classified as resistant to first and second line antibacterials.

We regarded coagulase-negative Staphylococci, *Corynebacterium sp*, *Micrococcus spp* and *Cutibacterium acnes* as possible skin contaminants and analyzed them separately. *Staphylococcus lugdunensis* was not included.

### Variables collected

Epidemiological data allowed stratification by age group, sex, hospital type (community *versus* university), and year of detection. “Early hospital-acquired” was defined as BSI between 2 and 5 days after hospitalization, whereas “late hospital-acquired” BSI were those occurring > 5 days after hospitalization. The remaining BSIs were labeled as community-acquired. The duration of hospitalization was calculated using hospital admission date and BSI date. ICU admission date was not available.

### Statistical analysis

The analytical plan had two steps: (1) to describe characteristics of BSI in different hospital acquisition scenarios (i.e., community *versus* early hospital-acquired *versus* late hospital-acquired); (2) to describe trends in pathogen distribution and resistance during the hospitalization using graphical descriptions and descriptive statistics. Characteristics depending on the hospitalization duration were compared with chi-square or Fisher test, as appropriate. The prevalence of a specific microorganism was calculated as the number of this microorganism over the total number of isolates. The prevalence of resistance (i.e., against first or second line antibiotics) was calculated as the number of resistant strains over the total number of this isolate. Changes in the prevalence during the hospitalization were assessed using the Cochrane–Armitage test. A sensitivity analysis excluding fungi was added. The data analysis for this paper was generated using (1) SAS software, version 9.4 (SAS Institute, Inc., Cary, NC), and (2) R version 3.6.1 (R Core Team (2020). R: A language and environment for statistical computing. R Foundation for Statistical Computing, Vienna, Austria. https://www.R-project.org/). p values < 0.05 were considered to be statistically significant. As the analysis was performed on anonymized non-genetic surveillance data, ethical consent was not required according to the Swiss law for research on humans.

## Results

**Figure 1:**
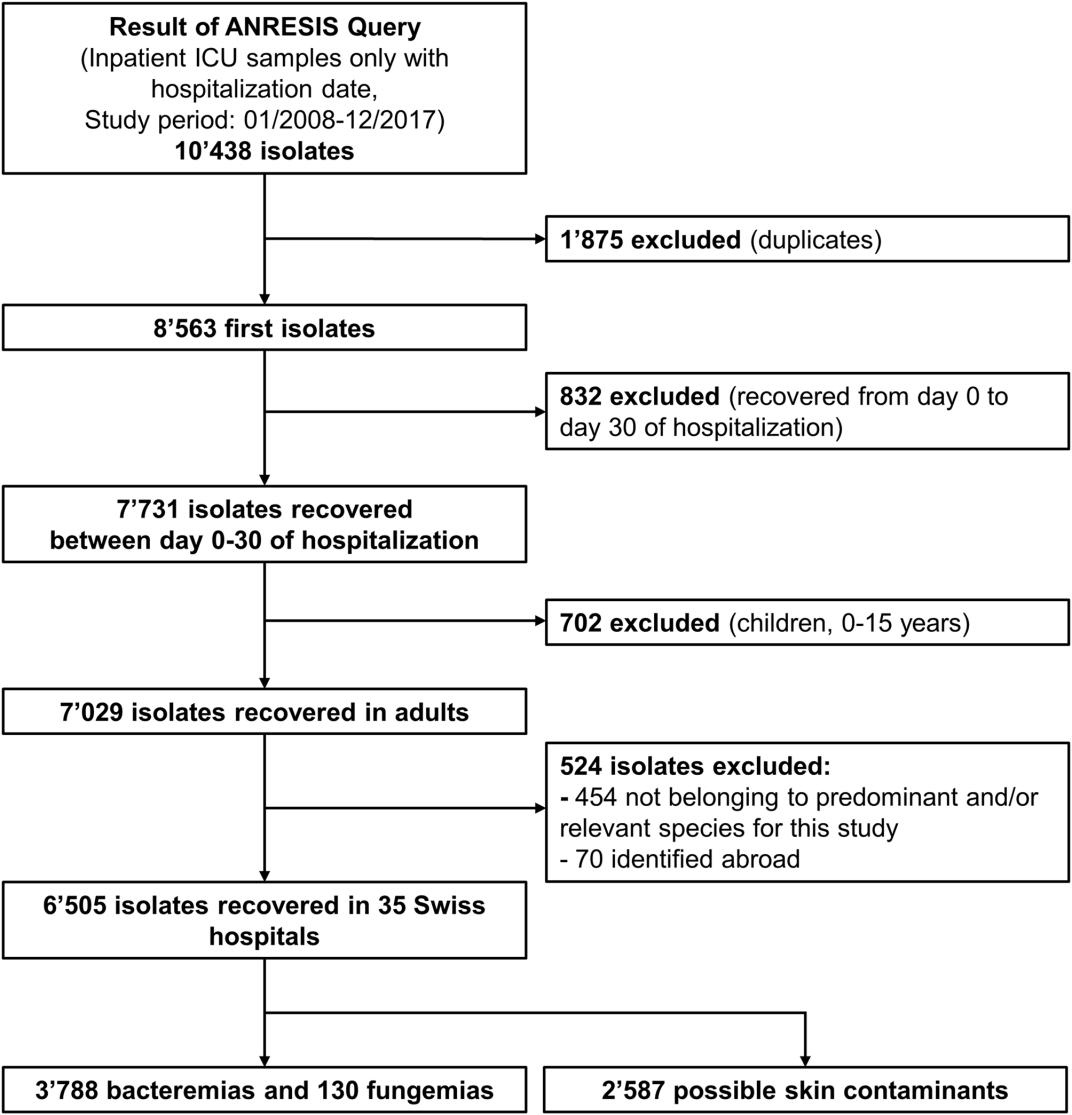
Flowchart of ICU-BSI included.

**Figure 2:**
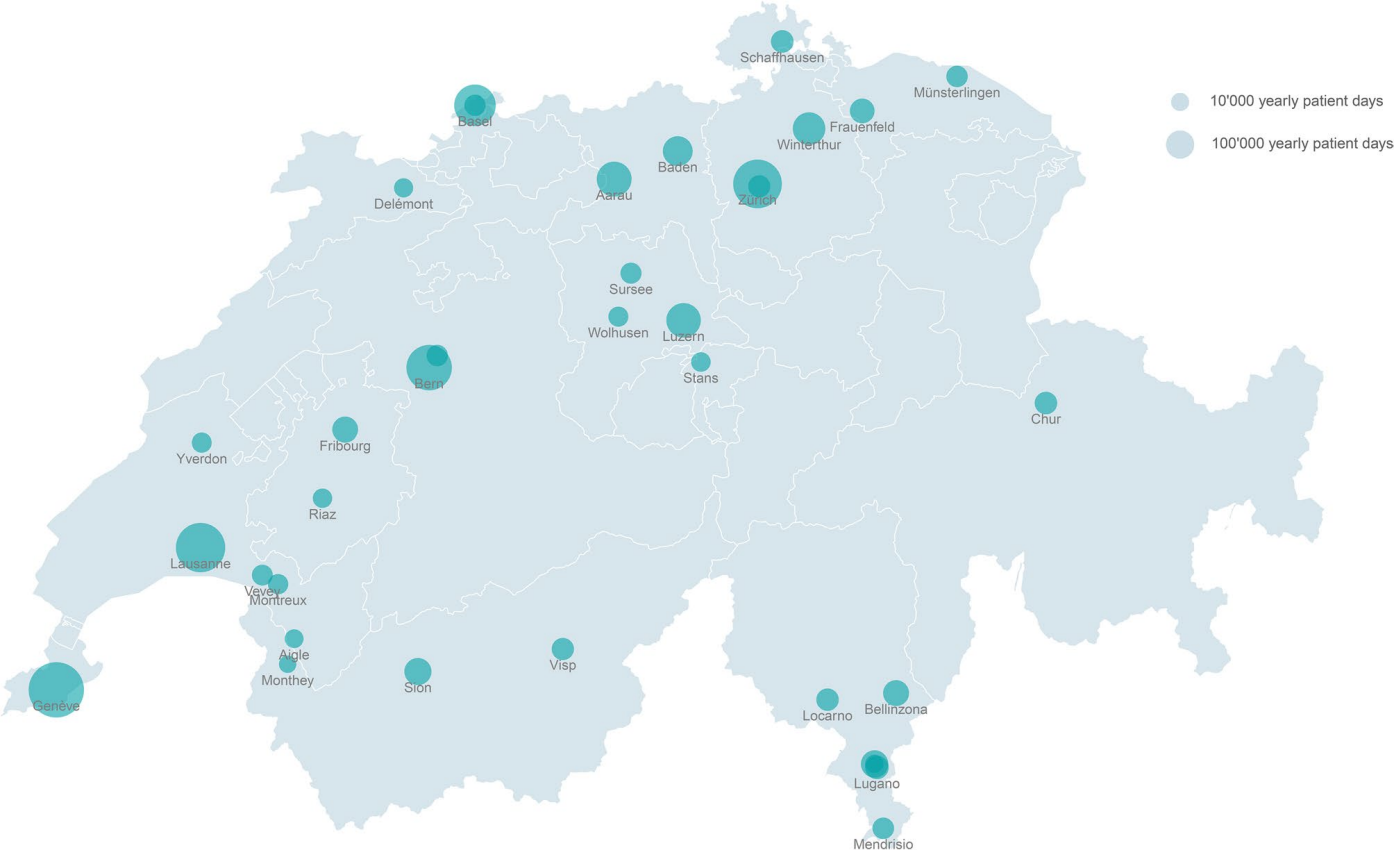
Swiss hospitals included in the study. The disk sizes are proportional to the hospitals’ annual patient days. The map was generated with R Version 3.5.2 and the raster package: https://cran.r-project.org/web/packages/raster/index.html.

From 2008 to 2017, we included data on 6505 ICU-BSIs from 35 Swiss hospitals (Figs. 1, 2). Possible skin contaminants were 2587.

After excluding possible skin contaminants, we observed 3788 bacteremias and 130 fungemias. BSIs were community-acquired in 2441 cases; whereas early hospital-acquired and late hospital-acquired cases amounted to 1377 and 2687, respectively. Late hospital-acquired BSIs were more frequently observed in male patients and the causative microorganisms were more frequently resistant against first line antibiotics (Table 1).

**Table 1:** Characteristics of BSI stratified by acquisition.

	Community-acquired (n = 2441)	Early hospital-acquired (n = 1377)	Late hospital-acquired (n = 2687)	p value
Age ≥ 60 years, n (%)	1729 (70.8)	932 (67.7)	1827 (68)	0.04
Male sex, n (%)	1511 (61.9)	962 (69.9)	1896 (70.6)	< 0.01
Hospital type, non-university hospital, n (%)	1594 (65.3)	951 (69.1)	1741 (64.8)	0.02
Year 2012–2017 (versus 2008–2011), n (%)	1146 (46.9)	744 (54)	1337 (49.8)	< 0.01
First line resistance (versus susceptible)	565 (23.1)	523 (38)	1530 (56.9)	< 0.01
Second line resistance (versus susceptible)^a^	37 (1.5)	28 (2.1)	112 (4.3)	< 0.01

Community acquired: 0–2 days after hospital admission. Early hospital-acquired: 2–5 days after the hospitalization. Late hospital-acquired: > 5 days after the hospitalization.

*ICU* intensive care unit, *n* number.

^a^135 missing values.

The most common identified microorganism was *Escherichia coli* (23.2%, 910), followed by *S. aureus* (18.7%, 734) and enterococci (13.1%, 515).

**Figure 3:**
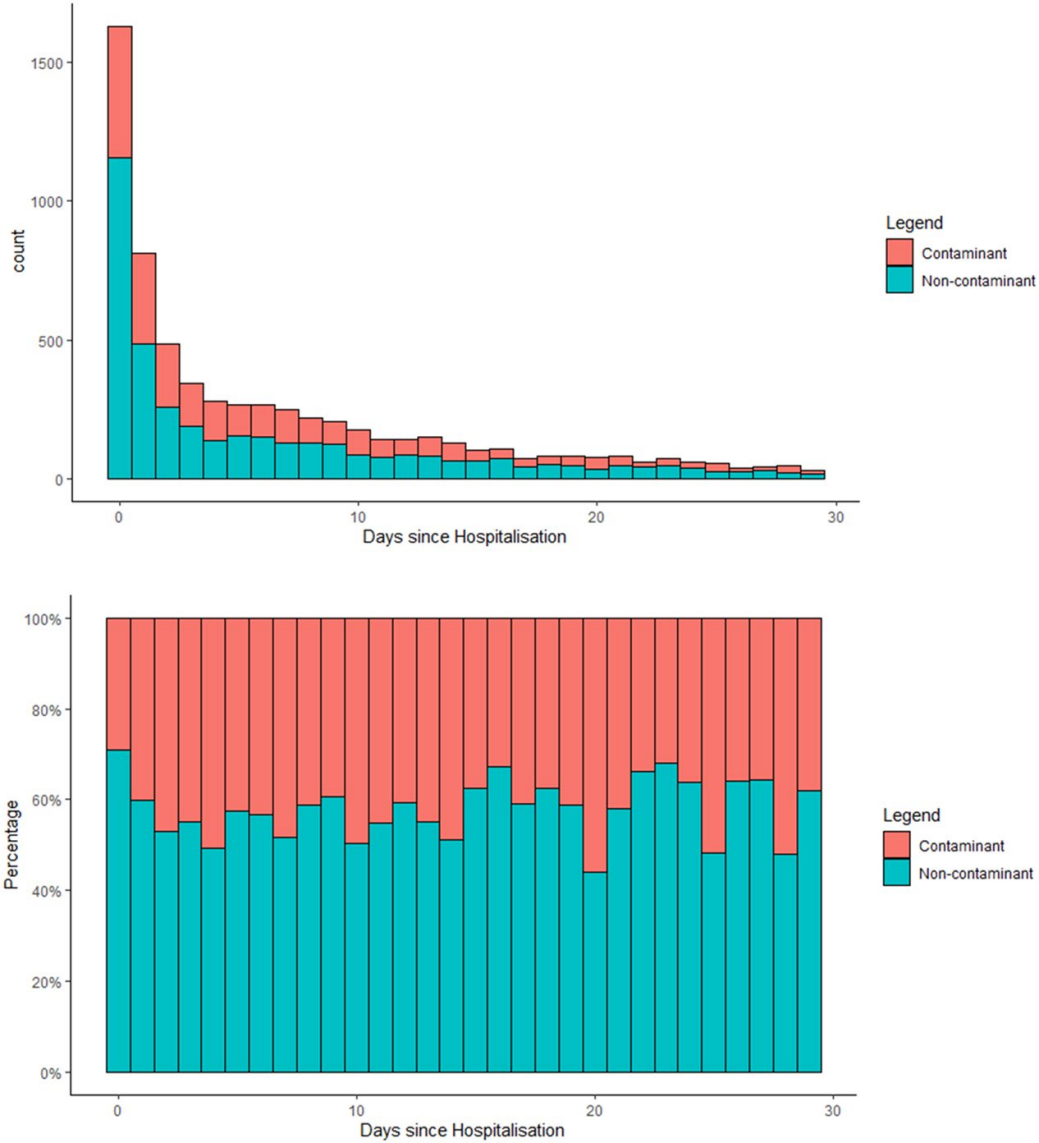
Number and proportion of microorganisms and possible skin contaminants relative to the hospitalization duration. Microorganisms included bacteria and fungi.

The number of microorganisms and possible skin contaminants observed during the study period are shown in the Fig. 3. The percentage of possible skin contaminants was low on day zero (29%, n = 473); after which the proportion of possible contaminants did not show a significant trend (p for trend = 0.18).

**Figure 4:**
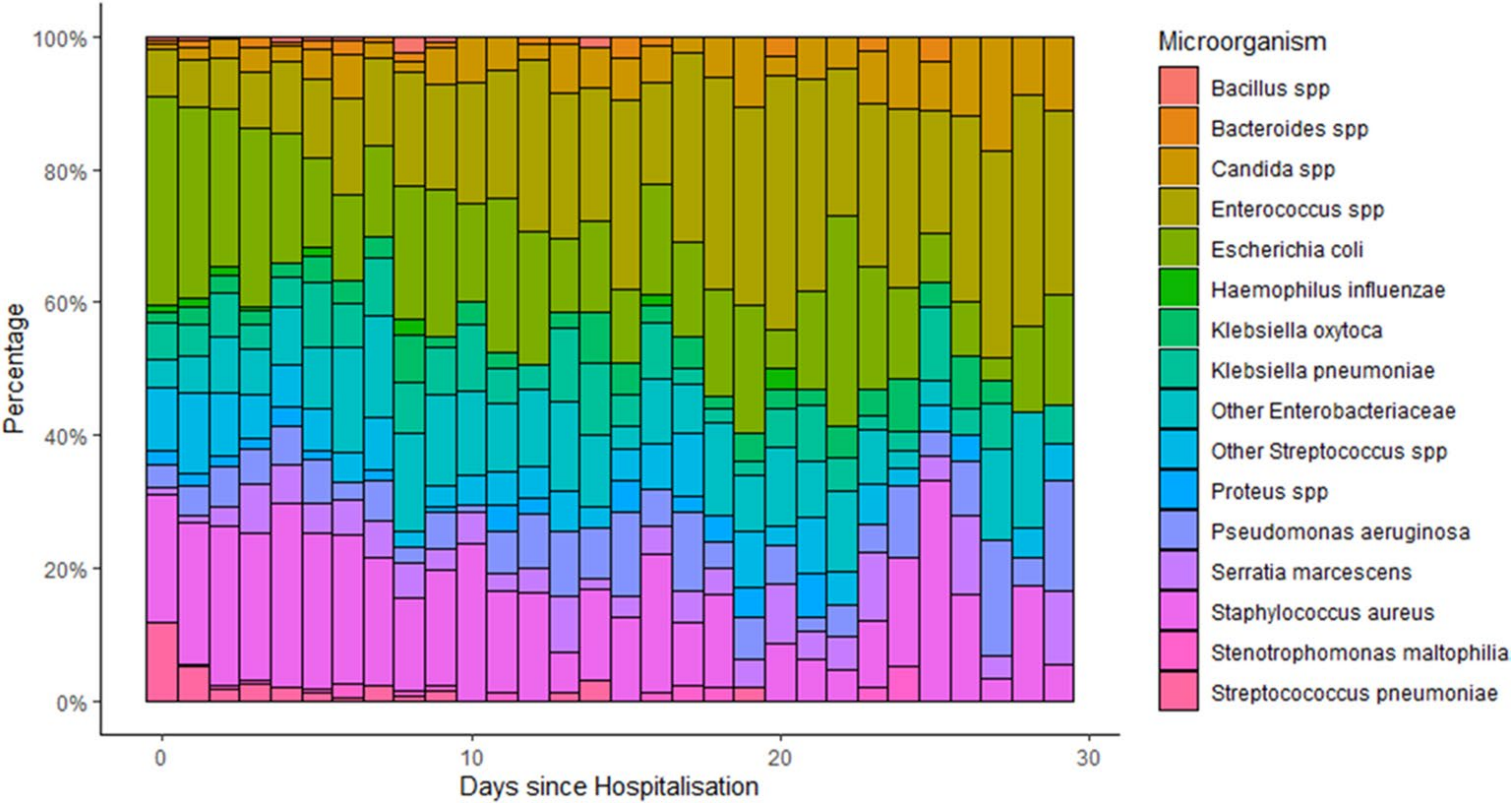
Distribution of microorganism observed in the ICU relative to the hospitalization duration. *spp* species.

The distribution of microorganisms during the hospitalization is shown in Fig. 4.

We observed an increasing trend for *Enterococci* from 7.1% on day zero to 28% on day 30 (p < 0.001) and for *Candida* spp. from 0.7 to 11.1% (p < 0.001). *E coli* decreased from 31.4% on day zero to 16.7% on day 30 (p < 0.0001) and *S. aureus* decreased from 19 to 5.6% (p < 0.0001). *Klebsiella pneumoniae* did not show a significant trend (p = 0.41).

**Figure 5:**
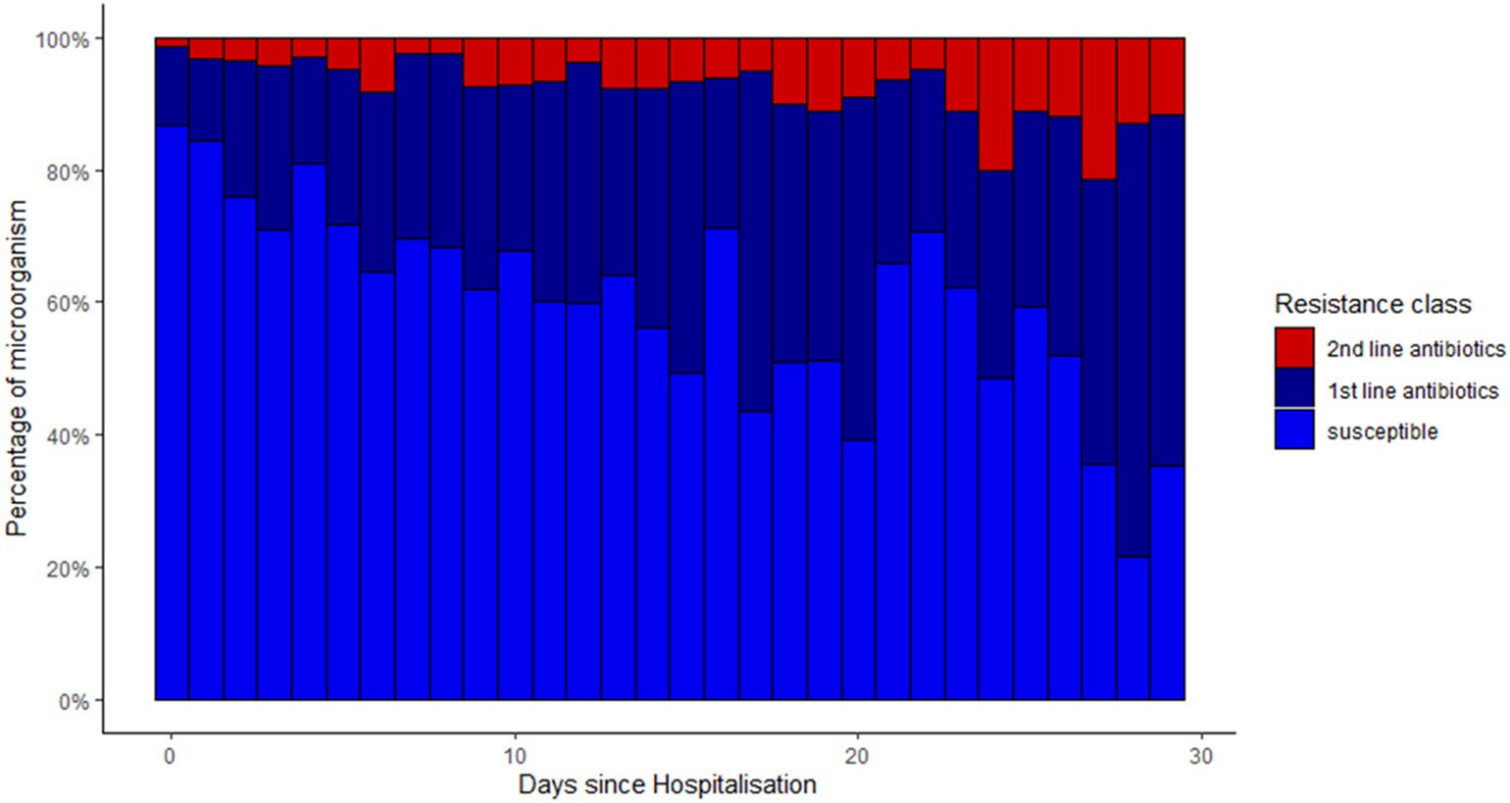
Antimicrobial resistance to first- and second-line antibiotics relative to the hospitalization. First-line antibiotic resistance (dark blue): ceftriaxone for gram-negative microorganisms, amoxicillin for Enterococci or oxacillin for *S. aureus*. Second-line antibiotic resistance (red): carbapenem for Gram-negative and vancomycin for Gram-positive microorganisms. Since antifungal agents were not empirically administered, fungi are shown (or included) here as resistant to 2nd line antibiotics.

Antimicrobial resistance to first-line antibiotics was 13.1% on day zero and then increased continuously to more than 60% on days 28–30 (p < 0.001, Fig. 5). Similar trends were observed excluding fungi from the analysis (p < 0.001, Supplementary Figure S1). Increasing trends were also observed for resistance to second-line antibiotics (p < 0.001).

## Discussion

Using a large microbiological database, we analyzed data on 6505 BSI episodes from 35 Swiss ICUs. Data in the literature on hospitalization duration and distribution of pathogens are scant. To our knowledge, only the EPIC I study showed rates of selected pathogens in the ICU setting depending on the number of hospital days before the study day^4^. However, this study (1) did not analyze data relative to the entire hospitalization and (2) did not *specifically* address this research question. We showed that the proportion of enterococcal BSI and resistant microorganisms against first- and second-line antibiotics increased substantially during the hospitalization. Moreover, we observed that possible contaminants showed the lowest proportion at hospital admission (i.e., day zero) but for the remainder of the hospitalization their proportions did not change.

Interestingly, we observed that the proportion of *Enterococci* increased during the hospitalization and accounted for almost 30% of all BSIs in late hospitalizations. It is conceivable that the exposure to antibiotics including the increasing use of cephalosporins in Switzerland^11^, the accumulation of complications and the often increasing case-severity during the hospitalization may predispose patients to develop enterococcal BSI. Although we did not investigate individual clinical patient data, our results may help clinicians in selecting empirical therapies for patients with long hospitalization duration.

Similarly, we observed that resistance against first or second line antibiotics increased linearly during the hospitalization. Clinicians and intensivists often apply a specific cut-off for assessing the acquisition of BSIs (i.e., community versus hospital-acquired). These cut-offs frequently guided the selection of antimicrobial therapies. For Gram-negative bacteremias, several authors selected a time cut-off of 5 days for comparisons between early and late onset following hospital admission^12^. In light of our results, simplistic recommendations based on a single time cut-off may not properly reflect the complex ICU-acquired BSI epidemiology. Therefore, regarding selection of empirical antibiotic therapy, an oversimplified “community” *versus* “hospital-acquired” classification based on duration of hospitalization should be discouraged.

Possible skin contaminants were low on the first day and then remained stable. This may be explained by a higher skill level in emergency medicine personnel (*versus* those on wards) when drawing blood cultures^13^. Another explanation for the higher proportion of possible contaminants during ICU stays (i.e., excluding the day zero) may be the collection of blood through intravascular catheters, which are more prone to contamination^14,15^. Trends of possible contaminants should be interpreted with caution: we did not perform a patient-based assessment for possible contamination and *possible* contaminants should not be considered as *proven* blood culture contaminations.

Our study has several limitations. First, we performed an analysis of a microbiological database and clinical data were unavailable to us (e.g., baseline comorbidities, reasons for admission, ICU admission date and reason, source of BSI and antibiotic treatment) and recommendations on BSI management could not be derived from this database. Second, Switzerland is a low-prevalence country regarding multidrug-resistant microorganisms and our data cannot be easily generalized to other countries. Third, we defined fungemic BSI episodes as resistant to first class antibiotics: formally, the epidemiology of fungemia cannot be equated with the epidemiology of bacteremia. However, we performed a sensitivity analysis excluding fungi which showed similar trends. Fourth, we performed simple descriptive analyses without adjustment for other confounders; moreover, we did not provide a more sophisticated risk prediction analysis and related score. Fifth, a selection bias could have been introduced when restricting the analysis to BSI with known acquisition relative to hospitalization duration. Finally, the raw number of ICU-BSI episodes decreased during the hospitalization and, therefore proportional trends should be interpreted with caution.

## Conclusions

Using data from a large national microbiological database we describe pathogen distribution and bacterial resistance in ICU-BSI during hospitalization. The proportion of enterococcal BSI, candidemia and resistant microorganisms against first and second line antibiotics increased during hospitalization.

### Ethics approval

As the analysis was performed on anonymized non-genetic surveillance data, ethical consent was not required according to the Swiss law for research on humans.

## Supplementary Information

Supplementary Information 1.

## Data Availability

The datasets used and/or analyzed during the current study are available from the corresponding author on reasonable request.
