# Supplementary material for: Distribution of pathogens and antimicrobial resistance in ICU-bloodstream infections during hospitalization: a nationwide surveillance study
Source: Sci Rep. 2021 Aug 19;11:16876. doi: 10.1038/s41598-021-95873-z (PMC8376881; doi:10.1038/s41598-021-95873-z)
Supplement: Supplementary file 1 — Supplementary Information 1. [file 41598_2021_95873_MOESM1_ESM.docx]

**Supplementary Figure 1: Antimicrobial resistance to first- and second-line antibiotics relative to the hospitalization excluding fungi.**

**
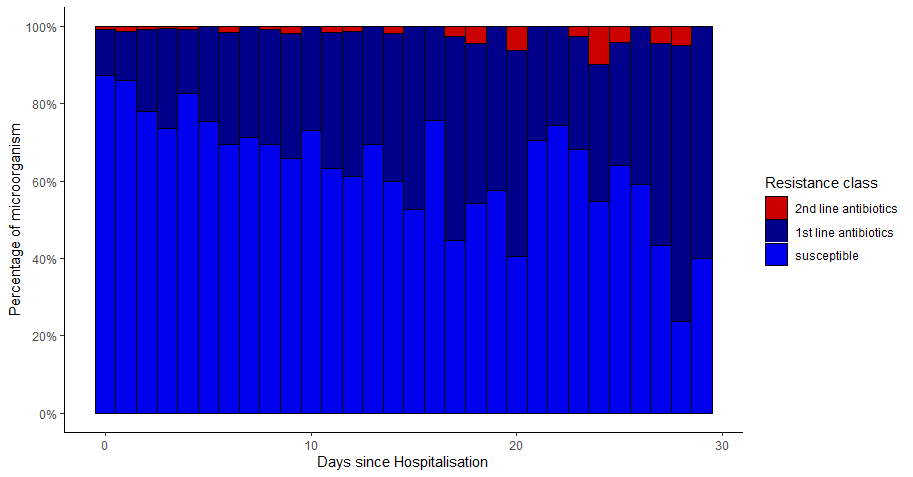
**

Notes. First-line antibiotic resistance (dark blue): ceftriaxone for gram-negative microorganisms, amoxicillin for Enterococci or oxacillin for *S. aureus*. Second-line antibiotic resistance (red): Carbapenem for Gram-negative and vancomycin for Gram-positive microorganisms.
